# The relationship between meaning of life, perceived social support, spiritual well-being and pain catastrophizing with quality of life in migraine patients: the mediating role of pain self-efficacy

**DOI:** 10.1186/s40359-023-01053-1

**Published:** 2023-01-23

**Authors:** Majid Yousefi Afrashteh, Mahya Abbasi, Mahsa Abbasi

**Affiliations:** 1 Department of Psychology, Faculty of Humanities, University of Zanjan, Zanjan, Iran; 2 Department of Psychology, Family Research Institute, Shahid Beheshti University, Tehran, Iran; 3 Department of Psychology, Faculty of Psychology and Educational Sciences, Shahid Beheshti University, Tehran, Iran

**Keywords:** Quality of life, Meaning of life, Perceived social support, Spiritual well-being, Pain catastrophizing, Pain self-efficacy, Migraine patients

## Abstract

**Background:**

Migraine is a neurological disease that has several physical and psychological complications, which is characterized by disability and impaired quality of life.

**Aims:**

The aim of this study was to explore the mediating role of pain self-efficacy in the relationship between meaning of life, perceived social support, spiritual well-being and pain catastrophizing with quality of life in migraine sufferers. The relationship between these factors with quality of life (QOL) was not fully explored in migraine patients.

**Method:**

This study was a correlational study of structural equations. Therefore, 300 patients with migraine who referred to one of the specialized neurological treatment centers in Zanjan in 2021 were recruited based on the inclusion criteria. Patients also completed the World Health Organization Quality of Life Scale (WHOQOL-BREF), Meaning in Life Questionnaire, Multidimensional Scale of Perceived Social Support, Spiritual Well-Being Scale, Pain Catastrophizing Scale, Pain Self-Efficacy Questionnaire. Finally, the hypotheses were then analyzed with correlation coefficient and path analysis method by using SPSS-26 and LISREL-10.2 programs.

**Results:**

The results of the present study showed that pain self-efficacy has a mediating role in the relationship between meaning of life and quality of life (B = 0.015), perceived social support with quality of life (B = 0.022), spiritual well-being with quality of life (B = 0.021), as well as pain catastrophizing with quality of life (B = − 0.015).

**Conclusion:**

According to the results of this study, by considering the role of self-efficacy of pain, it is possible to develop the programs to strengthen and improve the meaning of life, perceived social support, spiritual well-being and also reduce pain catastrophizing, in order to improve the quality of life of patients with migraine.

## Introduction

Migraine is a neurological disease and one of the most common and disabling type of chronic headache [1–3]. Therefore, the World Health Organization has mentioned migraine as one of the essential public health priorities [4]. The prevalence of migraine in the world that based on a review of 302 community-based studies is estimated 11.6% [5], and also the prevalence of it in the Iranian population is 14% [6]. Migraine peaks between the ages of eighteen and forty-four [7], and is more common in women [8]. This chronic disease is an intermittent, sudden, unilateral and pulsating headache that lasts about 4 to 72 h, and is associated with nausea and sensitivity to light and sound [9]. Genetic and environmental factors play roles in its occurrence [10].

When people are struggling with a physical illness, they simultaneously confront with psychological consequences; this fighting has a far-reaching effect on all aspects of patient’s lives that can lead to reduced quality of life [1, 3, 11, 12]. The World Health Organization refers to quality of life as the perception of individuals in relation to their goals, expectations, standards, concerns, and includes physical health, mental status, independence, social relationships, and personal beliefs [13]. If patients feel supported and safe in these cases, they can fight with more symptoms of the disease [10].

QoL is influenced by many factors, if these factors are improved, the quality of life in different aspects can increase in individual’s life [14]. One of the main elements in promoting people's satisfaction and quality of life is the meaning of life, which is a mental judgment and unique to each person [15–17]. Existential psychologists have argued that the experience of meaning in life lies at the heart of human existence [18]. Meaning is an important psychological resource both in situations of achievement and in critical situations [19]. In fact, having meaning in life can help patients identify their goals for life and encourage them to learn how to improve their lifestyle [20].

Since interpersonal relationships also have an effective role on the quality of life, therefore the study of psychosocial aspects of life in patients with migraine has particular importance [1, 15]. When faced with the challenges of a chronic illness, social support, as a protective factor, has crucial importance [21]. The effect of this variable can vary according to the source of support and perception of individuals accelerates the healing process [11, 22]. Perceived social support is a person's perception about the amount of support from the social network and the quality of support in stressful life situations [23]. This interaction provides material and psychological support, by establishing an empathic relationship and forming the safety network for the patient [24, 25]. In fact, it has an effective role not only in protecting against diseases but also in creating adaptation to diseases and increasing the effects of treatment in patients with chronic migraine [26].

Probably spirituality is one of the important aspects of well-being to deal with disease [27]. One way that can measure spirituality is the construct of spiritual well-being [28]. In the last two decades, the relationship between spiritual health and quality of life has been emphasized globally [29, 30]. Spiritual well-being as a strategy to deal with stressors has an effective role in reducing pain and increasing physical and psychological health in patients with cancer [31]. In other words, without a proper level of spiritual health, the function of other aspects of people's lives will be disrupted, and therefore it will not be possible to achieve the highest level of quality of life [32].

Catastrophizing is a psychological construct [33], that has been associated with impaired functioning and quality of life across a variety of chronic pain disorders [34]. Pain catastrophizing is defined as a negative cognitive-affective response to pain, and a tendency to exaggerate pain symptoms with feeling helpless [35, 36], which can increase perception of intensity of pain and emotional distress [37, 39]. The magnification can be a reflection of painful stimuli as a threatening subject, whereas helplessness may reflect the individual's perception of his or her disability to cope with painful stimuli [39, 40]. High levels of pain catastrophizing have more emotional reactions, in patients with migraine [39] and with chronic pain [41].

Living with chronic pain is associated with significant emotional stress [42], in this condition another important cognitive variable in coping with pain is self-efficacy, which refers to an individual's assessment of his or her ability to control behaviors [43]. Pain-related self-efficacy as a perceived ability is: (A) To continue normal daily functioning despite pain and, (B) Control and cope with pain symptoms [44]. In this regard, this variable like a shield increases effective cognitive beliefs, problem-solving ability and adaptation of patients with chronic pain [42]. Higher levels of pain self-efficacy in people experiencing chronic pain are associated with more positive outcomes [45].

The results of recent studies such as Majernikova and Obrocnikova [17] in cancer patients; Park et al.[46], Barsaei et al. [47], and Liu et al. [48] in patients with heart failure; showed a significant relationship between meaning of life and quality of life. Findings of DeMaria et al. [49] in patients with multiple chronic diseases; Costa et al. [25], and Kever et al. [21] in patients with multiple sclerosis; Ren et al. [50] in patients with chronic wounds; Qi et al. [51] in patients with type 2 diabetes; Aydın, and Demir [22], and Dun et al. [52] in cancer patients; indicate that there is a positive and significant correlation between social support and quality of life. Wysocka et al. [53] have shown that there is relationship between meaning of life, spirituality and quality of life in patients under the end-of-life care. According to researches of; Lee [54] in patients with lung cancer; Pilger et al. [28] in adults with hemodialysis; higher levels of spiritual well-being is associated with increasing the QOL scores in the physical, psychological, social relationships, and environmental aspects. Also Bai and Lazenby [55], that review 36 articles, were declare that a majority of studies reported positive correlation between overall spiritual well-being and quality of life.

According to the research of Alvarez-Astorga et al. [39] pain catastrophizing increases the symptoms of migraine in patients. Based on studies conducted by Kazi et al. [56] in patients with Chronic rhinosinusitis; De Carlo et al. [57] in young patients with Inflammatory bowel disease; Sewell et al. [33] in chronic illnesses; MackPeak et al. [58] in women with endometriosis; pain catastrophizing is associated with higher levels of pain that reduced quality of life. Research by Kalapurakkel et al. [45] showed that higher levels of pain self-efficacy are recognized as a protective psychological resource in patients experiencing chronic pain and are associated with better performance. Hashimato et al. [59] also indicated that self-efficacy in patients with Rheumatoid arthritis affects their quality of life. D’Amico et al. [60] in patients with Chronic Migraine concluded that self-efficacy and social support impact on quality of life. studies Chin et al. [61] in women with breast cancer, confirmed that self-efficacy significantly influence the quality of life. the researches of Mohajerani et al. [62]; and Hirata et al. [63] in patients have shown that self-efficacy can affects pain catastrophizing.

According to the research background in the world, the variables of the current study have not been studied simultaneously in migraine patients and most of researches have focused in other diseases. Accordingly, this study, in order to complete the gaps of previous researches and explore mediating role pain self-efficacy in quality of life of migraine patients, seeks to answer the question that is there a relationship between meaning of life, perceived social support, spiritual well-being and pain catastrophizing with quality of life in patients with migraine by mediator role of pain self-efficacy?

The meaning of life has a direct and indirect relationship (with the mediation of pain self-efficacy) with the quality of life of migraine patients.

The social support has a direct and indirect relationship (with the mediation of pain self-efficacy) with the quality of life of migraine patients.

The spiritual well-being has a direct and indirect relationship (with the mediation of pain self-efficacy) with the quality of life of migraine patients.

The pain catastrophizing has a direct and indirect relationship (with the mediation of pain self-efficacy) with the quality of life of migraine patients.

## Research methods

### Participants

The population of this research included patients with migraine who referred to one of the neurology clinics in Zanjan city from October 2021 to December. Diagnosis was made according to the criteria of the International Classification of Headache Disorders, third edition (ICHD-III beta, 2013) down to third-digit level (code 2.3) by a neurologist in headache diagnosis and management [64]. 350 participants were selected by convenience sampling method and according to Cochran's sample size formula. This formula suggested the number of 320 people for an approximate population size of 20,00 with an error level of 0.05 and a standard deviation of 0.5. The inclusion criteria included having migraines, being in the 25–45 age range, not taking psychiatric drugs, no experience of mourning in the past month before the survey, and giving informed consent to participate. These criteria were checked by self-reporting. The exclusion criteria included non-response to more than 15% of the questionnaire items and loss of appropriate cooperation conditions, such as illness. Finally, the data of 326 participants entered the final analysis. Of 326 participants, 33% were in the 25–30, 42% in the 31–35, and 18% in the 36–45 age groups. The age range of the participants was limited so that more accurate generalizations could be made. Also, 51% were man, and 58% had a university degree. More details are given in Table 1.

**Table 1:** Demographic statistics of the subjects

Variable	Frequency	Percent
Gender		
Man	159	51
Woman	167	49
Age		
25–30	108	33
31–35	147	45
36–45	71	22
Education		
Diploma or lower	132	40
Bachelor	109	33
Masters	40	12
Ph.D	35	10
Headache frequency days/month)		12 (3.85)*
Headache intensity (0–10)		5.2 (2.07)*
Headache duration (hours/attack)		6.14 (2.30)*

*Mean(standard deviation)

### Procedure

This cross-sectional study was conducted from October 30 to December 25. After obtaining the necessary permits and letters of introduction, participants were identified based on inclusion criteria. Research instruments were prepared on paper and online. The choice of answering on paper or online was determined by the participants and in both cases it was done in the presence of the research representative. In order to collect data, Zanjan's specialized brain and nerve treatment centers were referred, and eligible subject were asked to complete the questionnaires after reviewing the entry criteria. All methods were performed in accordance with the relevant guidelines and regulations. The questionnaires were initially distributed among 350 patients. After excluding 24 participants (participants who had more than 15% non-response items), the data of 326 patients were included in the final analysis. The response rate was finally 0.93. All rights of the participants were protected during this study. The procedures performed in the study involving human participants were according to the ethical standards of the National Research Committee. This study was approved by the Research Committee of The University of Zanjan. Participants completed a consent document before the survey and were allowed to leave the study at any time.

### Instruments

#### World Health Organization Quality of Life Scale (WHOQOL-BREF)

The questionnaire has been developed by a group of specialists in the WHO to assess people’s general quality of life and consists of four subscales: physical health, psychological health, social relationships, and environment domains that add up to a total score. The scale comprises 26 items scored on a 5-point Likert scale (1 = very bad to 5 = very good). After doing necessary calculations in each score domain, the resulting scores will range from 4 to 20, where 4 and 20 represent the worst and best states in that particular domain, respectively. The scores can be converted to a scale of 0–100, in which a higher score indicates a better condition. The scale’s validity was determined in the range of 0.46–0.67, and the reliability for the fourfold subscales and the total scale were determined between 0.73 and 0.89 based on Cronbach’s Alpha [65]. In Iran, Nejat et al. reported the validity and reliability of the scale at 0.45–0.83 and 0.88, respectively [66]. The reliability of this instrument in the present study was 0.77.

#### The Meaning in Life Questionnaire (MLQ)

The Meaning in Life Questionnaire was developed by Steger et al. to evaluate two aspects of meaning in life – i.e., the existence of meaning in life and the search for meaning in life [67]. The questionnaire comprises 10 items scored on a 7-point Likert scale (1 = completely incorrect to 7 = completely correct). The questionnaire’s minimum and maximum total scores are 10 and 70, respectively. A higher score on this scale indicates existence of meaning and valuable purposes in life. Steger et al. reported the reliability and validity of the subscale of the existence of meaning as 0.70 and 0.86, respectively, and the reliability and validity of the subscale of the search for meaning in life as0.73 and 0.87, respectively [67]. Moreover, Peimanfar et al. determined the reliability coefficient of the meaning in life questionnaire at 0.89 using Cronbach’s Alpha formula [68]. According to the research of Mesrabadi et al. the meaning in life questionnaire enjoyed favorable construct and diagnostic validity in Iran [69]. The reliability of this instrument in the present study was 0.82.

#### The Multidimensional Scale of Perceived Social Support (MSPSS)

Zimet et al. developed this scale that consists of 12 items and 3 subscales. The items are scored on a 5-point Likert scale (1 = completely disagree to 5 = completely agree) [70]. The three subscales include the perceived support of family, the perceived support of friends, and the perceived support of significant others. The scale’s total score is obtained by summing up the scores given to the items. The minimum and maximum scores are 12 and 60, respectively. A higher score indicates more significant perceptions of social support. Zimet et al. reported its Cronbach’s Alpha coefficient and test-rest reliability at 0.85–0.91 and 0.72–0.85, respectively [69]. In Iran, Salimi et al. reported the scale’s reliability for the perceived support of family, friends, and significant others as 0.86, 0.86, and 0.82, respectively, using Cronbach’s Alpha formula [71]. The reliability of this instrument in the present study was 0.75.

#### The Spiritual Well-Being Scale (SWBS)

Ellison and Paloutzian developed this scale that evaluates the perceived quality of spiritual life in three domains: religious well-being, existential well-being, and overall spiritual well-being using 20 items [72]. SWBS was translated into Farsi by Abhari et al. [73] in Iran and its psychometric properties were analyzed and confirmed. The Persian version was implemented in this research. Items with odd numbers are related to the religious well-being subscale and assess one’s satisfactory relationship with God. On the other hand, the items with even numbers are related to the existential subscale and assess one’s purposefulness and life satisfaction. The items are scored on a 6-point Likert scale (1 = completely agree to 6 = completely disagree) [74]. The minimum and maximum total scores for spiritual well-being are 6 and 120, respectively. A higher score in the questionnaire indicates enhanced spiritual well-being. Ellison and Paloutzian reported the reliability of the religious well-being, existential well-being, and the total scale as 0.91, 0.91, and 0.93, respectively, using Cronbach’s Alpha [72]. In Iran, Ansari et al. reported the reliability of the spiritual well-being scale as 0.88, using Cronbach’s Alpha, and confirmed the acceptable validity of the scale using the confirmatory factor analysis [75]. The reliability of this instrument in the present study was 0.84.

#### The Pain Catastrophizing Scale (PSC)

Sullivan et al. developed this questionnaire to evaluate pain catastrophizing thoughts and behaviors. The scale has been designed to evaluate various aspects of pain catastrophizing and better understand the impacts of the pain catastrophizing mechanism on the experience of pain [76]. The scale comprises 13 items with three subscales: rumination, exaggeration, and helplessness. Examinees are asked to rate their pain-related thoughts and feelings on a continuum that ranges from 0 (never) to 4 (always). The total score is obtained by summing up the scores given to each item with minimum and maximum scores of 0 and 52, respectively. A lower score indicates less pain catastrophizing, while a higher score shows more pain catastrophizing. Meyer et al. reported the Cronbach’s Alpha reliability of rumination, exaggeration, helplessness, and the total scale as 0.88, 0.67, 0.89, and 0.92, respectively [77]. The questionnaire was first translated to Persian by Sajadian et al., who implemented it on a sample of women with chronic backache and determined its reliability coefficient at 0.93 [78]. The reliability of this instrument in the present study was 0.76.

#### The Pain Self-Efficacy Questionnaire (PSEQ)

Nicholas developed this questionnaire to evaluate the pain self-efficacy of patients with chronic pain [79]. The scale is a self-report instrument with 10 items where each item assesses the patients’ evaluation of their abilities to perform a set of activities despite the existence of pain. The items are scored according to a 7-point Likert scale (0 = I cannot do it all to 6 = I cannot do it completely). The total score ranges between 0 and 60, and a higher score indicates a higher sense of self-efficacy against chronic pain. Nicholas determined the scale’s reliability at 0.92, using Cronbach’s Alpha formula [79]. Moreover, Latifian et al. reported the Cronbach’s Alpha coefficient as 0.93 [80]. The reliability of this instrument in the present study was 0.88.

### Statistical analysis

SPSS v.26 (IBM) and LISREL v10.2 were used for data analysis. Descriptive analyses, including mean and standard deviation and Pearson correlation matrix with SPSS and path analysis, were performed using LISREL. Although in large samples, normality is less critical, in this study, the indices of Skewness and kurtosis were examined. As shown in Table 2, these indices are between − 1 and 1 for all variables, so the data distribution is normal, and there is no problem with using Pearson correlation and path analysis. Path analysis with ordinal data was conducted using the diagonally weighted least squares method (WLSMV). The model fit indices were Chi-square statistics, Chi-square/df, Root Mean Square Error of Approximation (RMSEA), Comparative Fit Index (CFI), Tucker-Lewis Index [TLI, also known as the Non-normed fit index (NNFI)], Goodness of Fit Index (AGFI) and Adjusted Goodness of Fit Index (AGFI). The model was judged as having a good fit when the overall picture of fit indices indicated good fit and excellent if all of them indicated well fit: RMSEA ≤ 0.05, CFI and TLI ≥ 0.95, and WRMR < 0.90 (36). Likewise, a significant PCLOSE (*p* < 0.05) indicates that RMSEA > 0.05 (and therefore, it is not a good model).

**Table 2:** Descriptive statistics for research variables and correlation coefficient between them

variable	M	SD	Correlation matrix				
			1	2	3	4	5
1. Meaning of Life	35.32	7.82	–				
2. Social Support	38.23	8.06	0.12^*^	^–^			
3. Spiritual Well-Being	66.38	9.89	0.09	0.11^*^			
4. Pain Catastrophizing	28.16	5.04	− 0.35^**^	− 0.28^**^	-0.11^*^		
5. Pain Self-Efficacy	31.73	5.79	0.20^**^	0.25^**^	0.21^**^	− 0.23^**^	
6. Quality of Life	56.73	11.02	0.30^**^	0.34^**^	0.28^**^	− 0.51^**^	0.31^**^

M = mean, SD = standard deviation

^*^p < 0.05; **p < 0.01

## Results

Table 2 shows the descriptive statistics including mean and standard deviation for meaning of life, social support, spiritual well-being, pain catastrophizing, pain self-efficacy and quality of life in migraine sufferers. Also, Pearson correlations are reported to determine the relationship all between variables. The mean and standard deviation of quality of life are 56.73 and 11.02, respectively. The correlation coefficient of quality of life with meaning of life was 0.30, with social support was 0.34, with spiritual well-being was 0.28, with pain catastrophizing was − 0.51, and with pain self-efficacy was 0.31. All these coefficients are significant at the level of 0.05 or 0.01. More details are shown in Table 2.

Table 3 shows the direct, indirect, and total effects for the relationship of the variables in the model to pain self-efficacy and quality of life. According to the results of this table, meaning of life (β = 0.11), social support (β = 0.16), spiritual well-being (β = 0.18), pain catastrophizing (β = − 0.37) and pain self-Efficacy (β = 0.12) have a significant (t > 1.96) direct effect in the variance of quality of life. Also, meaning of life (β = 0.12), social support (β = 0.18), spiritual well-being (β = 0.17), and pain catastrophizing (β = − 0.12) have a significant (t > 1.96) direct effect in the variance of pain self-efficacy.

**Table 3:** Path coefficients for direct, indirect and total effects between variables

Dependent	predictors	Direct effect	t-value	Indirect effect	t-value	Total effect	t-value
Quality of Life (R^2^ = 0.37)	Meaning of Life	0.11	2.30	0.015	2.57	0.12	2.65
	Social Support	0.16	3.48	0.022	2.03	0.19	2.79
	Spiritual Well-Being	0.18	3.97	0.021	2.00	0.20	2.35
	Pain Catastrophizing	− 0.37	− 7.55	− 0.015	− 1.99	− 0.39	− 6.80
	Pain Self-Efficacy	0.12	2.58	–	–	0.12	2.58
Pain Self-Efficacy (R^2^ = 0.13)	Meaning of Life	0.12	2.22	–	–	0.12	2.22
	Social Support	0.18	3.30	–	–	0.18	3.30
	Spiritual Well-Being	0.17	3.17	**–**	–	0.17	3.17
	Pain Catastrophizing	− 0.12	− 2.06	–	–	− 0.12	− 2.06

**Fig. 1:**
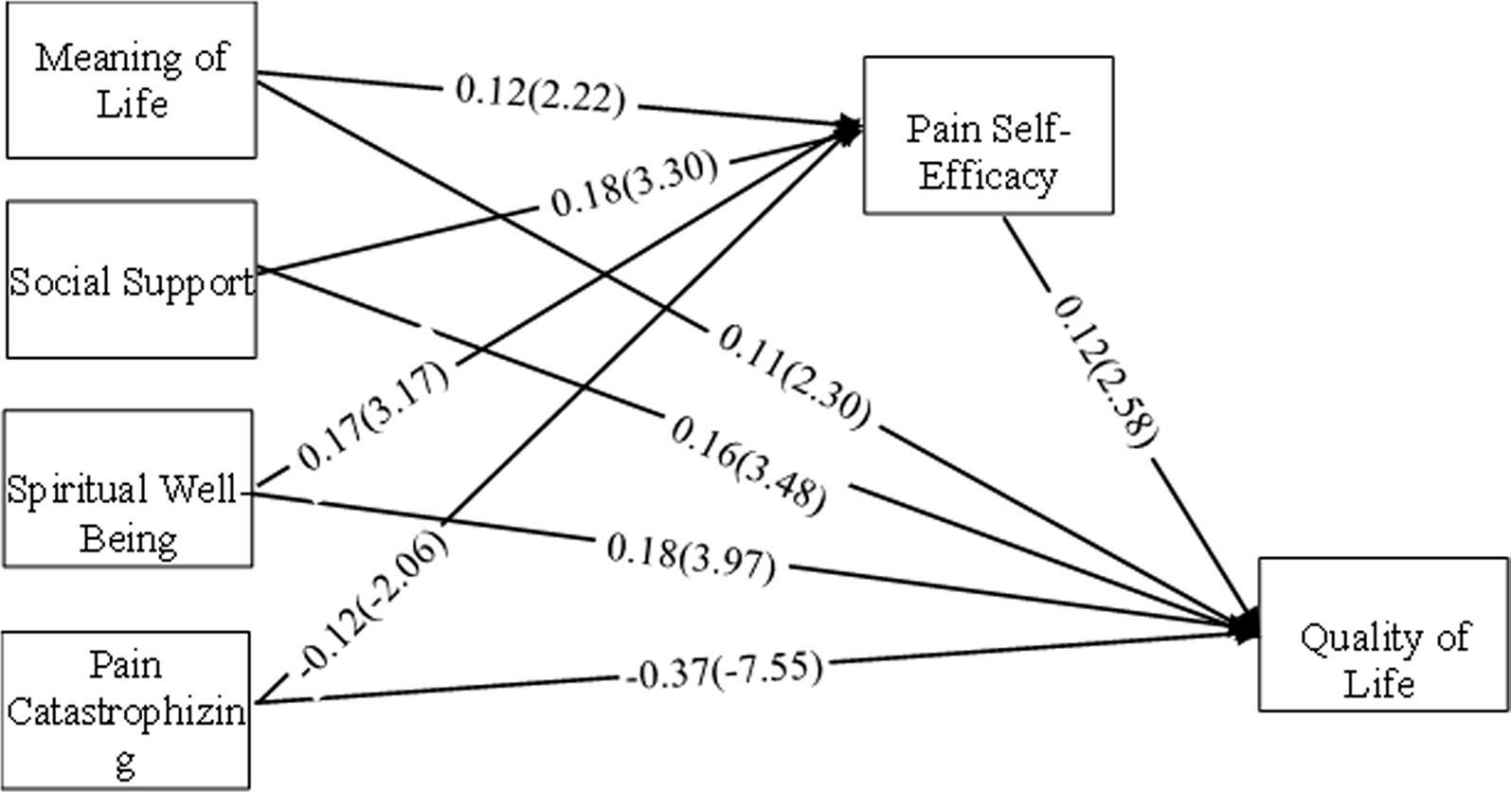
Standard estimate (and t-value) for relationship between variable

According to the results of Table 3, the mediating role of pain self-efficacy in the relationship between meaning of life, social support, spiritual well-being, pain catastrophizing with quality of life is significant. Therefore, in addition to the direct effect of meaning of life, social support, spiritual well-being, pain catastrophizing on quality of life, their indirect effect was also confirmed by mediation alone of pain self-efficacy. Figure 1 shows the relationships obtained from path analysis with the standard parameter index and the t-value (in parentheses) on the paths.

The goodness-of-fit indices reported in Table 4 show that the analyzed model has an excellent fit.

**Table 4:** The goodness of Fit Indices for the Models

Index	χ2	*p* value	df	χ2/df	RMSEA	CFI	AGFI	NFI	IFI
Value	1.08	0.29	1	1.08	0.02	0.95	0.95	0.93	0.96

χ2/df: χ2to the degree of freedom index; RMSEA: root mean square error of approximation; CFI: comparative fit index; AGFI: Adjusted goodness fit index; NFI: Normed Fit Index; IFI: Incremental Fit Index

## Discussion and conclusion

The aim of this study was to explore the mediator role of pain self-efficacy in the relationship between meaning of life, perceived social support, spiritual well-being and pain catastrophizing with quality of life in migraine sufferers. Therefore, 300 patients with migraine were recruited and hypotheses were tested by using path analysis method.

In examining the first hypothesis of the study, the findings showed that the meaning of life has a direct significant relationship with quality of life (B = 0.11). Also, the meaning of life is indirectly significantly related to quality of life through mediation of pain self-efficacy (B = 0.015), results of the present study are consistent with findings of Majernikova and Obrocnikova [17]; Park et al. [46], Barsaei et al. [47]; Liu et al. [48]. In explaining these findings, it can be stated that the search for meaning is a stressful process which high levels of meaning search, will lead to less adaptation, while high levels of meaning presence will lead to more adaptation and life satisfaction symptoms [81]. In such conditions, self-efficacy of pain as a protective factor, creates the belief that despite the presence of pain, it can function optimally [45, 82]. Low self-efficacy leads to failure and a sense of lack of control over life events, and people believe that any attempt to find meaning is futile, while patients with high self-efficacy, see illness as a challenge rather than a threat [48]. For overcome to this challenge, by confidence in their abilities and changing the lens through which they view their life events, they try to reconstruct the meaning of their lives, that ultimately improves their quality of life in different dimensions [81].

In examining the second hypothesis of the research, the results confirm the hypothesis that perceived social support has a direct significant relationship with quality of life (B = 0.16). Also, indirectly perceived social support through mediation of pain self-efficacy has a significant relationship with quality of life (B = 0.022). These results are consistent with findings of De Maria et al. [49]; Costaet al. [25], and Kever et al. [21]; Ren et al. [50]; Kucukakca et al. [24]; Qi et al. [51]; Aydın, and Demir [22]; and Dun, et al. [52]. In explaining this result, due to humans are inherently social and need a secure and social environment to survive, illness are serious threats to active community interaction and confidence to competencies [21, 24]. Perceived social support can improve health and quality of life by increase self-care, adherence to the doctor's advice, compliance to treatment, changing lifestyle, increasing awareness and access to information of disease [83, 84]. In fact, perceived social support effectively reduces adverse physiological reactions to the disease, and by strengthening their ability and sense of self-efficacy, helps them become better equipped to cope with the disease [12]. This high self-efficacy and patient confidence in their ability, carries decline in avoidance of disease and facilitates adherence to treatment [60].

In examining the third hypothesis of the research, the findings of the present study showed that spiritual well-being has a direct significant relationship with quality of life (B = 0.18). Also, spiritual well-being is indirectly significantly related to quality of life through mediation of pain self-efficacy (B = 0.021). Consistent with the results of the present study, the findings of Wysocka et al. [53]; Lee [54]; Pilger et al. [28]; and Bai and Lazenby [55], they have shown that there is a significant positive correlation between spiritual well-being and quality of life. In explaining the relationship between spiritual well-being and quality of life, it can be stated that spirituality by creating hope and a sense of meaning in life, can help them cope better in difficult situations and improve their quality of life [29]. Spiritual well-being is associated with pain self-efficacy in patients with chronic pain [85]. In other words, when patients have high self-efficacy, they have efficient beliefs regarding treatment [86]. It can also be said that persons with higher scores of spiritual well-being, pay more attention to their mental health and more inclined to adapt to stressful stimuli of life [28]. Finally it can help to them increase their quality of life [28].

In examining the fourth hypothesis of the research, the obtained results indicate that pain catastrophizing has an indirect significant relationship with quality of life (B = − 0 /37). Pain catastrophizing also has a significant indirect relationship with quality of life through pain self-efficacy mediation (B =− 0.015). Therefore the results of the present study with the research of Alvarez-Astorga et al. [39]; Sewell et al. [33]; McPack et al. [58]; De Carlo et al. [57]; Hayashi et al. [87]; Galvez Sanchez et al. [88]; Kazi et al. are consistent [56] And all have reported significant inverse relationships between pain catastrophizing and quality of life. In explaining these findings, it can be said that pain perception is a complex phenomenon and has cognitive, emotional, behavioral and motivational dimensions that affect each person differently [33]. Patients overestimate their pain by having catastrophizing beliefs of rumination and magnification, on the other hand, the emergence of feelings of helplessness [40]. Also low levels of pain self-efficacy and exaggerated negative evaluations can create a vicious cycle [33]. By increasing the pain, feeling of helplessness gradually increases and then pain management becomes more difficult, which in turn reduce quality of life, especially in patients with migraine [33].

### Research limitations

The method of the present study is correlational and the cross-sectional research design, and also data collection has been done in a limited period of time. Therefore, it does not allow us to define causal relationships. This study was performed on migraine patients in Zanjan, therefore the generalizability of the results is limited. Another limitation is that the research was based on patient perception and also the simultaneous implementation of multiple tools and multiple items may have affected the precision of the response. Also in the present study, the effect of gender has not been controlled.

### Research suggestions

Accomplishment similar research using qualitative methods as well as experiments that have more power in identifying causal relationships. Also, the present study should be performed on a wider sample of patients with different age, educational, occupational and socio-economic status it is also recommended in different cultures to increase the generalizability of the findings. Since men and women are different in their needs, such as the need for social affiliation, it is better to consider gender in research.

### Practical suggestions

This research provides more and deeper knowledge about the variables that affect the quality of life. Therefore, it can facilitate the design and development of intervention strategies. Therefore, health care professionals should evaluate the quality of life in patients with migraine by considering the meaning of life, perceived social support, spiritual well-being and pain catastrophizing with emphasis on pain self-efficacy for successful treatment planning and control treatment methods. So that patients can cope better with the disease and thus help increase their quality of life.

## Data Availability

The datasets during and/or analyzed during the current study available from the corresponding author on reasonable request.
